# Comparison of the cuff pressure of a TaperGuard endotracheal tube and a cylindrical endotracheal tube after lateral rotation of head during middle ear surgery: A single-blind, randomized clinical consort study

**DOI:** 10.1097/MD.0000000000006257

**Published:** 2017-03-10

**Authors:** Eunkyung Choi, Yongmin Park, Younghoon Jeon

**Affiliations:** a Department of Anesthesiology and Pain Medicine, Yeungnam University College of Medicine; b Department of Anesthesiology and Pain Medicine, School of Medicine; c Department of Anesthesiology and Pain Medicine, School of Dentistry, Kyungpook National University, Daegu, Republic of Korea.

**Keywords:** cuff, endotracheal tube, lateral rotation, pressure

## Abstract

**Background:**

Positional change affects the cuff pressure of an endotracheal tube (ETT) in treacheally intubated patients. We compared the cuff pressure of a TaperGuard ETT and a cylindrical ETT after lateral rotation of head during middle ear surgery.

**Methods:**

Fifty-two patients aged 18–70 years underwent a tympanomastoidectomy under general anesthesia were randomly allocated to receive endotracheal intubation with cylindrical (group C, n = 26) or TaperGuard ETTs (group T, n = 26). After endotracheal intubation, the ETT cuff pressure was set at 22 cmH_2_O in the neutral position of head. After lateral rotation of head, the cuff pressure was measured again and readjusted to 22 cmH_2_O. In addition, the change of distance from the carina to the tip of the ETT was measured before and after the positional change. The incidence of cough, sore throat, and hoarseness was assessed at 30 minutes, 6 hours, and 24 hours after surgery.

**Results:**

There was no difference in demographic data between groups. After lateral rotation of head, the cuff pressure significantly increased in group T (11.9 ± 2.3 cmH_2_O) compared with group C (6.0 ± 1.9 cmH_2_O) (*P* < 0.001). The incidence of a cuff pressure >30 cmH_2_O was higher in group T (96.2%) than in group C (30.8%) (*P* < 0.001). In addition, the degree of displacement of an ETT was greater in group T (11.0 ± 1.7 mm) than in group C (7.2 ± 2.6 mm) (*P* < 0.001). The overall incidences of postoperative sore throat, hoarseness, and cough at 30 minutes, 6 hours, and 24 hours after surgery were comparable between two groups.

**Conclusion:**

The cuff pressure was higher in the TaperGuard ETT than in the cylindrical ETT after positional change of head from neutral to lateral rotation. In addition, after a positional change, the extent of displacement of ETT was greater in the TaperGuard ETT than in the cylindrical ETT.

## 1 Introduction

The inflation of the endotracheal tube (ETT) cuff is very useful to prevent aspiration of contaminated substances into lung past ETT and leakage of gas during positive pressure ventilation. However, excessive inflation of ETT cuff frequently causes tracheal mucosal damage, which can increase the incidence of sore throat, hoarseness, and coughing after surgery. When a sealing cuff pressure is >30 cmH_2_O, insufficient perfusion of tracheal mucosa occurs. Therefore, a sealing cuff pressure of 20–30 cmH_2_O is clinically important for a proper seal and prevention of tracheal injury in tracheally intubated patients.

A cuff pressure is affected by several factors such as the physiological character of an ETT, the use of nitrous gas, and positional change during general anesthesia^.^^[1–4]^ In the tracheal cartilages, the cricoids cartilage is the only round shape, followed by C-shaped tracheal cartilages. Therefore, a positional change causes displacement of an ETT in the trachea, which may affect the cuff pressure.^[5,6]^ The physical characteristics of the ETT cuff may also affect the cuff pressure.^[7]^ The shape of a classic ETT cuff is cylindrical, whereas that of a newly developed TaperGuard ETT is distally tapered. It was demonstrated that a newly developed TaperGuard ETT is more effective in providing a sealing effect than a cylindrical ETT in an in vitro study.^[8,9]^ However, recently, it was reported that the cuff pressure of a TaperGaurd ETT significantly increased after a positional change from the supine to the lateral flank position, compared to that of a cylindrical ETT.^[10]^

The previous study showed that lateral rotation of head resulted in a greater increase of a tracheal mucosa pressure than the extended or flexed position.^[11]^ In addition, lateral rotation of head toward the side of ETT fixation caused displacement of the ETT away from the carina in adult patients.^[2]^ Therefore, this study was performed to evaluate the effect of lateral rotation of head on the cuff pressure of TaperGaurd ETT, compared to a cylindrical ETT during middle ear surgery.

## 2 Methods

### 2.1 Patients and exclusion criteria

The present study was approved by the Ethics Committee of Kyungpook National University Hospital (KNUH 2016-02-017-001) and an informed written consent was obtained from all patients. This study was registered in the ClinicalTrials.GOV (NCT02797938). A total of 52 patients with American Society of Anesthesiologists physical status classification I–III and aged 18–70 years for a tympanomastoidectomy under general anesthesia were included. Exclusion criteria were history of difficult intubation, limited neck movement, respiratory diseases, and body mass index >35 kg/m^2^. The written informed consent from all patients was obtained.

### 2.2 Anesthesia and data collection

**Figure 1:**
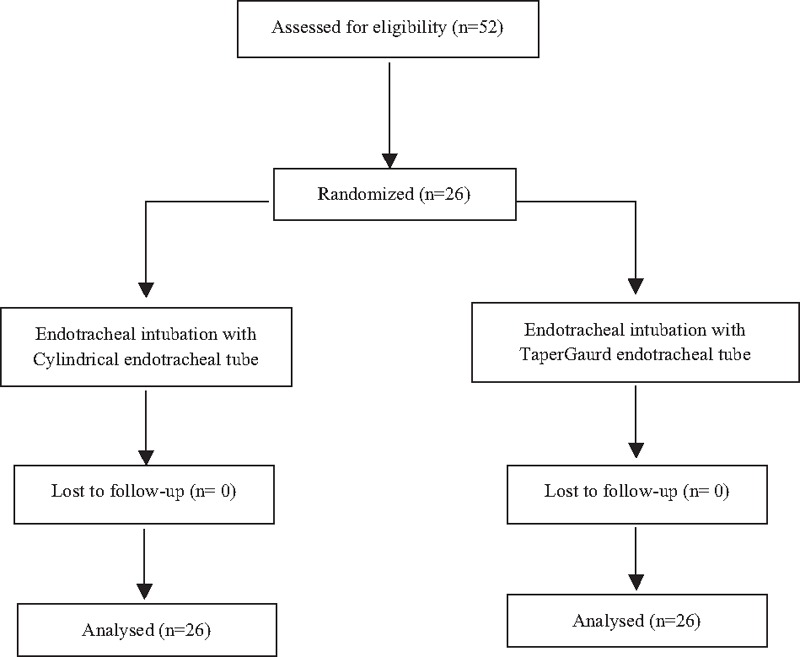
Flow diagram of the study.

Patients were allocated into two groups using computer-generated randomization assed by a physician who was not involved for perioperative care (Fig. 1). The patient in group C (n = 26) were intubated with a cylindrical-shaped ETT (Unomedical, Kedah, Malaysia) and the patient in group T (n = 26) was intubated with a TaperGuard ETT (Covidien, Mansfield, OH). The group allocations were informed to the attending anesthesiologist before anesthesia. Tracheal intubation was performed with a size of inner diameter 7.5 mm ETT for males and 7.0 mm ETT for females by the same anesthesiologist. The anesthesiologist measured cuff pressure and distance from the ETT tip to the carina during the study period.

Premedication was not administered. In the operating room, the patients were monitored with noninvasive arterial blood pressure, pulse oximetry, and electrocardiography. General anesthesia was induced with propofol 2 mg/kg, and rocuronium 0.8 mg/kg. Endotracheal intubation was done with a direct laryngoscope and the tip of the ETT was placed at 4 cm above carina using a fiberoptic bronchoscope. The ETT was fixed with tape on the contralateral side to operation. The cuff of the ETT was inflated with air and the cuff pressure was initially adjusted at 22 cmH_2_O using a manometer (Mallinckrodt Medical, Hennef, Germany). Head was laterally rotated to the opposite side of the surgical site and set at 45°. After rotation of head the cuff pressure was measured again and readjusted to 22 cmH_2_O. The distance from the ETT tip to the carina was recorded again using a fiberoptic bronchoscope. Anesthesia was maintained with 1.5–3.0 sevoflurane with 50% oxygen in air and with a target concentration of remifentanil set at 2–3 ng/mL. Volume-controlled ventilation was set at a tidal volume of 8 mL/kg and respiratory rate of 10–12 cycle/min to maintain the end-tidal carbon dioxide tension from 30 to 35 mm Hg.

The incidence of hoarseness, sore throat, and cough was assessed at 30 minutes, 6 hours, and 24 hours after surgery by a study-blinded anesthesiologist. The primary outcome was the cuff pressure after rotation of head. The secondary outcomes were the displacement of the ETT tip from the carina after lateral rotation of head and incidence of airway morbidity after surgery.

### 2.3 Sample size

A preliminary study using 15 volunteers showed that an increase of the cuff pressure of the TaperGuard ETT in the rotation of head was 9 ± 2.3 cmH_2_O (mean ± standard deviation). In this study, a 30% difference in the mean cuff pressure with the lateral rotation of head between the two ETTs was considered to be significant. Therefore, the minimum sample size of 23 patients per group was needed with a significance level of 0.01 (α = 0.01) and a power of 90% (β = 0.10). Considering 10% drop out rate, 26 patients in each group were needed.

### 2.4 Statistical analysis

Statistical analysis was performed using statistical software (SPSS, version 23.0 for Windows; SPSS, Chicago, IL) and used Student's *t*-test, chi-squared test, or Fisher's exact test, as appropriate. *P* < 0.05 was considered statistically significant. Data were expressed as mean ± SD or number (%).

## 3 Results

A total of 52 patients completed this study (Fig. 1). Patient demographic data and intraoperative data were comparable between two groups (Table 1). After lateral rotation of head, the cuff pressure significantly increased in group T compared with group C (35.7 ± 2.4 vs 30.0 ± 1.9; *P* < 0.001) (Table 2). After lateral rotation of head, the degree of displacement of the ETT tip was greater in group T than in group C (11.0 ± 1.7 mm vs 7.2 ± 2.6 mm; *P* < 0.001) (Table 2). The incidence of a cuff pressure >30 cmH_2_O in the position of lateral head rotation significantly increased in group T compared with group C (96.2% vs 30.8%; *P* < 0.05) (Table 3). The overall incidence of hoarseness, sore throat, and cough at 30 minutes, 6 hours, and 24 hours after surgery was comparable between two groups.

**Table 1:**
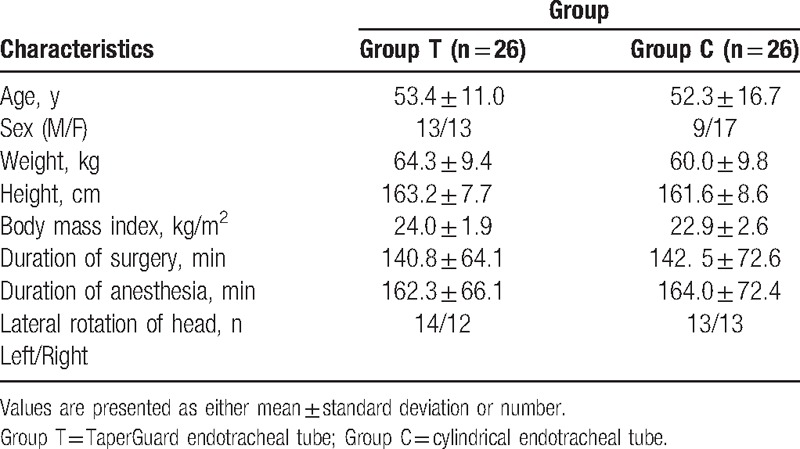
Patient characteristics and intraoperative data.

**Table 2:**
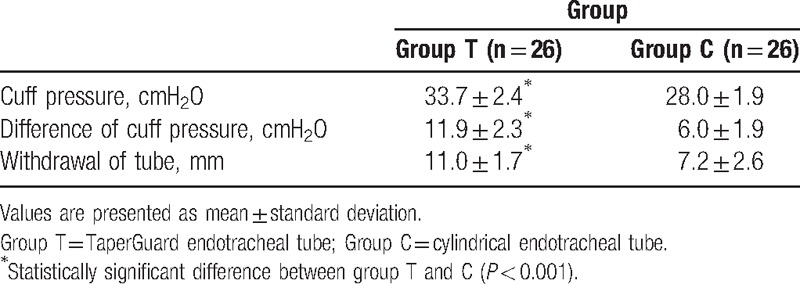
Cuff pressure and withdrawal of a tracheal tube after a positional change of head from neutral to lateral rotation.

**Table 3:**
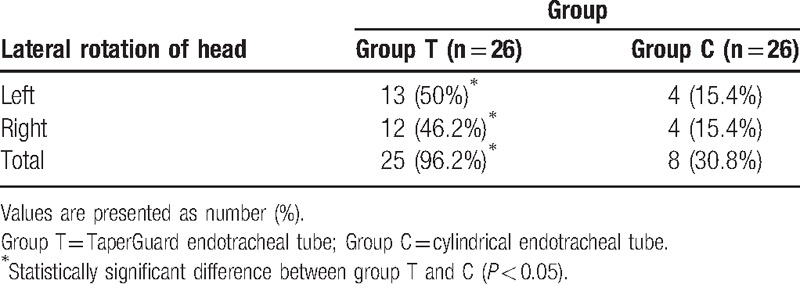
The cuff pressure >30 cmH_2_O in lateral rotation of head.

## 4 Discussion

In this study, the cuff pressure in the TaperGuard ETT significantly increased after lateral rotation of head compared with the cylindrical ETT. In addition, the extent of displacement of the ETT was greater in the TaperGuard ETT than in the cylindrical ETT after a positional change.

The excessive pressure of the ETT cuff caused the airway complications including rupture of trachea during mechanical ventilation, sore throat, hoarseness, and coughing after surgery.^[12–15]^ It was demonstrated that the ETT cuff pressure >30 cmH_2_O resulted in impairment of blood flow and histological lesions of the tracheal mucosa in the preclinical and the clinical studies.^[16,17]^ The ETT cuff pressure can be influenced by several factors such as patient factors (tracheal diameter), anesthetic factors (nitrous oxide), factor associated with a ETT (compliance and shape of a ETT cuff), and surgical factors (position and laparoscopic surgery).^[1,18–20]^

The diameter of the cylindrical ETT cuff is 120%–150% of the internal tracheal diameter. When inflated in the trachea, longitudinal folds or channels can be formed, leading to leakage of air or fluid.^[7]^ However, a TaperGuard ETT cuff has a diameter lesser than a cylindrical ETT cuff. Therefore, a cylindrical ETT has more folds or channels to ensure air seal than a TaperGuard ETT. It was demonstrated that a TaperGuard ETT is more effective for prevention of microaspiration and pneumonia during mechanical ventilation than a cylindrical ETT.^[9,21]^

In the present study, a cuff pressure in the lateral rotation of head significantly increased in the TaperGuard ETT compared with the cylindrical ETT. In addition, the incidence of a cuff pressure >30 cmH_2_O significantly increased in the TaperGuard ETT compared with the cylindrical ETT. In the previous study, it was demonstrated that the cuff pressure was higher in the TaperGuard ETT than in the cylindrical ETT after a positional change from neutral to lateral flank position although there was no difference in the degree of tube displacement between them.^[10]^ Therefore, it was suggested that the geometry of the ETT may be responsive for the significant difference in the intracuff pressure between TaperGuard and cylindrical ETTs because there is no difference of cuff compliance between them.^[10]^

The previous study demonstrated that the head rotation toward the tube fixation side significantly displaced the tube tip away from the carina, whereas head rotation to the non-fixation side resulted in a migration of the ETT in an unpredictable manner.^[2]^ In the present study, the ETT was fixed with a tape on the contralateral side to operation and after lateral rotation of head toward the tube fixation side proximal migration of the ETT occurred, which is consistent with the previous study.^[2]^ Boyle's law is a gas law stating that the pressure and volume of a gas have an inverse relationship at a constant temperature. In the present study, the cephalad migration of the ETT occurred after a positional change, which may place the ETT cuff adjacent to a less compliant airway structure including cricoids cartilage. The extent of the cephalad migration of the ETT is greater in the TaperGuard ETT than in the cylindrical ETT, which may be partly responsible for an significant elevation of the cuff pressure in the TaperGuard ETT compared with the cylindrical ETT.

In this study, there are several limitations. First, this is a single-blinded randomized study. Blinding was impossible. Therefore, a study unblinded anesthesiologist performed tracheal intubation and collected data during anesthesia, which can be a source of bias. Second, it was reported that inner diameter of the subglottis and upper trachea is less in a south Indian population than in a western population.^[22]^ The present study was conducted in an only Asian population at a single center, which limited the ability to extrapolate the results beyond the selected population. A multicenter study is needed to verify the utility of a TaperGuard ETT in the surgeries requiring a positional change of head and neck. Third, in this study, there were no differences in the incidences of postoperative airway complications such as sore throat, hoarseness, and cough between two groups. The cuff pressure in the TaperGuard ETT significantly increased in the lateral rotation of head, compared with the cylindrical ETT. After a positional change, we adjusted the cuff pressure to 22 cmH_2_O, which can explain the reason that the incidences of postoperative airway morbidity were comparable between two groups. However, our findings regarding the incidence of postoperative airway complications should be considered within the context of this study because the sample size was relatively small to detect a difference in airway morbidity. Further studies are required to investigate the effect of the shape of the ETT cuff on airway morbidity after surgery.

In conclusion, the head movement from neutral to lateral rotation can lead to a greater increase of the cuff pressure in the TaperGuard ETT than in the cylindrical ETT. In addition, the proximal migration of the ETT is higher in the TaperGuard ETT than in the cylindrical ETT.
